# Novelty during a late postacquisition time window attenuates the persistence of fear memory

**DOI:** 10.1038/srep35220

**Published:** 2016-10-13

**Authors:** Cynthia Katche, Micol Tomaiuolo, Guido Dorman, Jorge H. Medina, Haydee Viola

**Affiliations:** 1 Instituto de Biología Celular y Neurociencias, Facultad de Medicina “Dr Eduardo De Robertis” (UBA-CONICET), UBA, Paraguay 2155, 3 piso, Buenos Aires (C1121ABG), Argentina; 2 Departamento de Fisiología, Facultad de Medicina, UBA, Paraguay 2155, 7 piso, Buenos Aires (C1121ABG), Argentina; 3 Departamento de Fisiología, Biología Molecular y Celular Dr Hector Maldonado, Facultad de Ciencias Exactas y Naturales, Universidad de Buenos Aires, Buenos Aires (C1428EGA), Argentina

## Abstract

Learning to avoid threats in the environment is highly adaptive. However, sometimes a dysregulation of fear memories processing may underlie fear-related disorders. Despite recent advances, a major question of how to effectively attenuate persistent fear memories in a safe manner remains unresolved. Here we show experiments employing a behavioural tool to target a specific time window after training to limit the persistence of a fear memory in rats. We observed that exposure to a novel environment 11 h after an inhibitory avoidance (IA) training that induces a long-lasting memory, attenuates the durability of IA memory but not its formation. This effect is time-restricted and not seen when the environment is familiar. In addition, novelty-induced attenuation of IA memory durability is prevented by the intrahippocampal infusion of the CaMKs inhibitor KN-93. This new behavioural approach which targets a specific time window during late memory consolidation, might represent a new tool for reducing the durability of persistent fear memories.

Memories, including those which are painful or fear-inducing, are crucial for our lives and encompass the essence of who we are. They are quite important for our day-to-day functioning and hence for our quality of life. However, sometimes fear learning is maladaptive, generating persistent memories with an excessive fear and anxiety. Current therapies for fear-related disorders involve pharmacological or behavioural manipulations of long-lasting fear memories, and rely mainly on reconsolidation and extinction processes. However, extinction-based exposure therapy has limited efficacy^1^. For instance, immediate or delayed extinction procedures induce a reduction in aversive memories that are context-dependent and short-lived^2^,^3^. In contrast, when extinction training is given after the retrieval of a traumatic experience in healthy volunteers, a reduction of the original fearful memory is found^4^. This finding paralleled those obtained in rodents^5^, (but see ref. 6). However, it will be desirable to induce fear attenuation without the need to submit the individuals to the retrieval of the fear experience.

We and others have demonstrated the existence of a novel phase, happening around 12 h after acquisition, specifically involved in the persistence but not the formation of fear long-term memory (LTM)^7^,^8^. This phase depends on *de novo* protein synthesis in the hippocampus and amygdala and is controlled by dopaminergic inputs^9^. These findings might open an opportunity to generate new treatments to attenuate the persistence of fear memories.

We have previously reported that a robust retrograde amnesia of IA memory formation can be induced by novelty^10^ when is presented during the early stage of memory consolidation. Thus, on the basis of the above findings we reasoned that subjecting rats to a novel environment late after acquisition of a one trial IA learning task could selectively attenuate the maintenance of the mnemonic trace without interfering with its formation. This behavioural approach that targets a late and specific time window while consolidation is still in process represents a promising non pharmacological procedure for reducing the durability of persistent memories.

## Results

### Exposure to a novel OF exploration 11 h post-training impairs IA-LTM persistence

**Figure 1:**
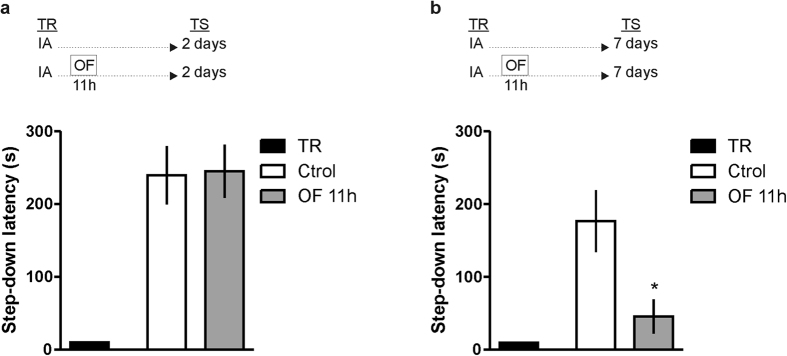
Exposure to a novel OF exploration 11 h post-training impairs IA-LTM persistence but not LTM formation. Rats were subjected to IA training (Ctrl) or to IA training plus a novel OF 11 h (OF 11 h) after IA training. Data are expressed as mean ± SEM of training (TR) or test (TS) session step down-latency at 2 (**a**) or 7 days (**b**) after IA training. *p < 0.05, Ctrl vs. OF 11 h group; Student’s t test, n = 10–12 per group.

**Figure 2:**
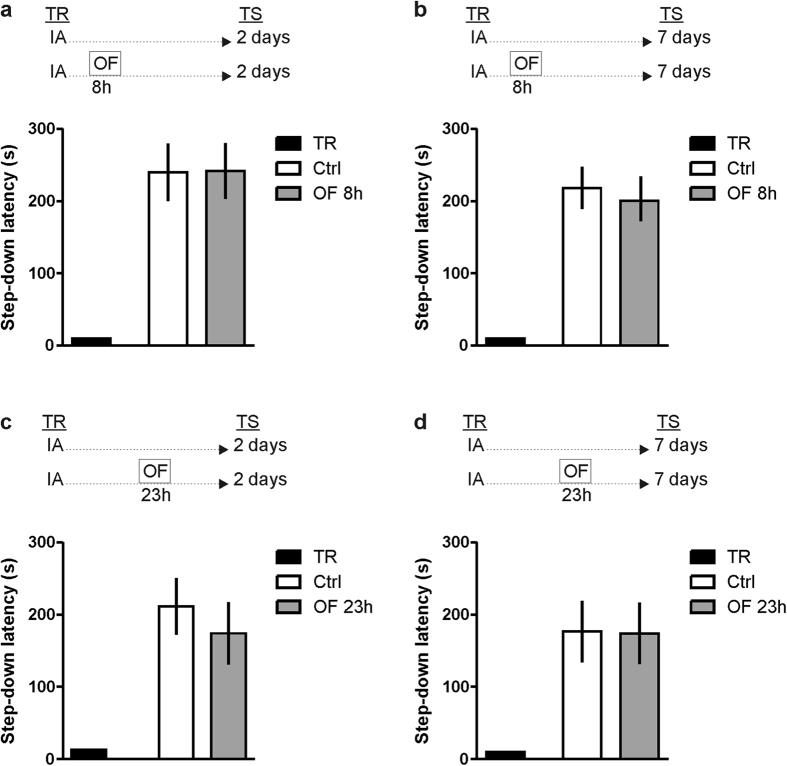
A novel OF exploration performed 8 or 23 h after training do not affect IA-LTM persistence. Rats were subjected to IA training (Ctrl) or to IA training plus a novel OF 8 h (OF 8 h; **a**,**b**) or 23 h (OF 23 h; **c**,**d**) after IA training. Data are expressed as mean ± SEM of training (TR) or test (TS) session step down-latency at 2 (**a**,**c**) or 7 days after IA training (**b**,**d**). Student’s t t.est, n = 8 per group.

The IA is a fear-motivated associative learning task that is hippocampus-dependent and acquired in a single and brief training session^11^. Exposure to an OF 11 h after IA training did not affect memory retention performance of the avoidance task when evaluated 2 days after training (Fig. 1a). In contrast, when an independent group of animals was tested 7 days after IA training we found an impairment in memory retention (Fig. 1b, p < 0.05 in comparison to the Control IA-trained group of rats that were not exposed to OF; Student’s t test, n = 10–12). This effect was observed during a critical time window after training because no altered retention scores were seen when IA-trained rats were exposed to a novel environment 8 or 23 h after training and tested 2 or 7 days thereafter (Fig. 2). These findings indicate that exposure to a novel environment late after training selectively attenuates, in a time-dependent manner, the persistence of IA LTM storage without altering memory formation.

### OF requires to be novel in order to impair IA-LTM persistence

**Figure 3:**
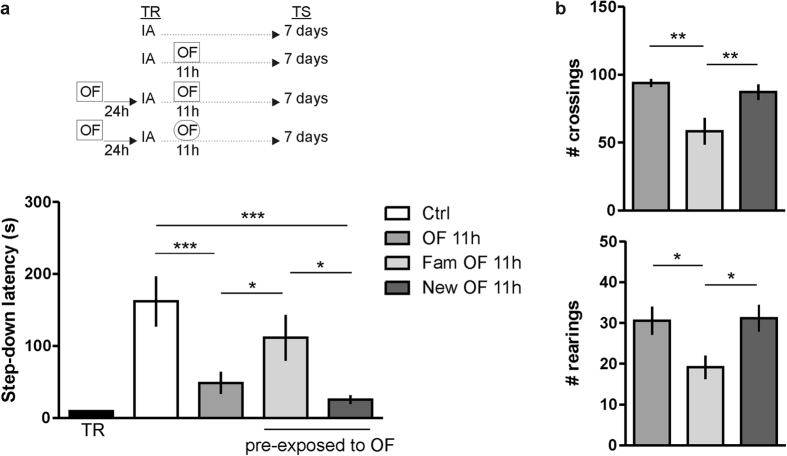
OF requires to be novel in order to impair IA-LTM persistence. (**a**) Animals were exposed to a single OF session 11 h post IA training (OF 11 h) or to an additional 30 min square OF session the day before IA training (pre-exposed to OF). Rats explored the square familiar OF (Fam OF 11 h) or a round shape novel OF (New OF 11 h) 11 h after IA training. Data are expressed as mean ± SEM of training (TR) or test (TS) session step down-latency at 7 days after IA training. *p < 0.05, vs. Fam OF 11 h group, ***p < 0.001 vs. Ctrl; Newman-Keuls test after ANOVA, n = 9–14 per group. (**b**) Bar graph represents the total number of quadrant crossings (Top) or rearings (Bottom) during 5 min in the OF 11 h, Fam OF 11 h and New OF 11 h groups. Data are presented as mean ± SEM. *p < 0.05; **p < 0.01, vs Fam OF 11 h group, Newman-Keuls test after ANOVA, n = 9–14 per group.

Is the attenuating effect of the novel environment on memory persistence due to the perception of novelty? To determine whether novelty is a key factor in reducing the durability of IA LTM, rats were exposed for 30 min to a square OF 24 h prior to the IA training. Animals were then exposed again to the square OF (Fam OF group) or to a round shape novel OF (New OF group) 11 h after IA training and tested for memory retention 7 days after. As shown in Fig. 3a, the Fam OF group did not exhibit any deficit (p > 0.05, compared to Ctrl group, n = 12–13). As before, animals subjected to a novel OF at 11 h posttraining had a clear-cut impairment in retention performance 7 days after training (Fig. 3a, p < 0.001 compared to Ctrl rats; Newman-Keuls test after ANOVA, n = 13–14). This amnesic effect induced by a novel OF was also seen when rats were exposed to the square OF the day before the IA training (Fig. 3a, p < 0.001 New OF 11hs compared to Ctrl rats; Newman-Keuls test after ANOVA, n = 9–13). Figure 3b shows, in addition, that rats subjected to a 2nd exposure of the same OF performed less crossings and rearings than rats that explored the OF for the 1st time or rats that experienced a different OF during the 2nd exposure. (p < 0.01 for crossing, p < 0.05 for rearings; Fam OF vs. other groups, Newman-Keuls test after ANOVA, n = 9–14). This implies that in the 2nd exposure to the same OF the animals recognized it as being familiar and, as a consequence, they explored it short time^12^.

### Novel OF impairs IA-LTM persistence through a CaMK-dependent mechanism

**Figure 4:**
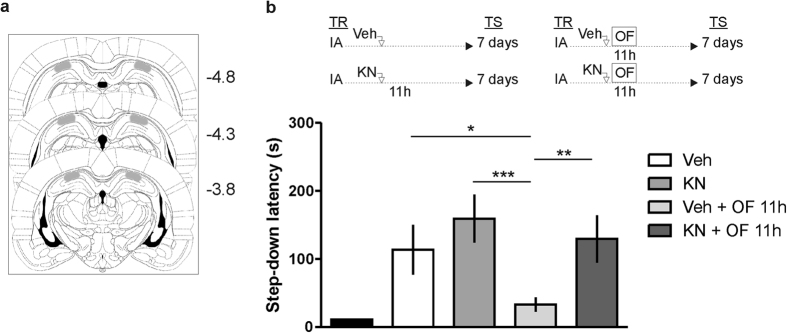
Novel OF impairs IA-LTM persistence through a CaMK dependent mechanism. (**a**) Schematic representation of rat brain sections at three rostrocaudal planes (AP: −3.8, −4.3, −4.8 mm, from bregma) taken from the atlas of Paxinos and Watson (1997). In stippling, the extension of the area reached by the infusions in the dorsal hippocampus (CA1). (**b**) Animals not exposed to OF were bilaterally infused with vehicle (Veh) or KN-93 (KN) into CA1 region of the dorsal hippocampus, 11 h after IA training as control groups. Animals exposed to a novel OF 11 h after IA training received intra CA1 infusions of either vehicle (Veh + OF 11 h) or KN-93 (KN + OF 11 h) 15 min before OF. Data are expressed as mean ± SEM of training (TR) or test session (TS) step down-latency at 7 days after IA training. *p < 0.05; **p < 0.01, ***p < 0.001, vs Veh + OF 11 h, Newman-Keuls test after ANOVA, n = 11–15 per group.

What are the molecular events required for attenuating fear LTM storage induced by novelty? Given that the detection of spatial novelty is associated with an activation of CaMKs in the dorsal hippocampus and that CaMKs are required for memory formation of OF habituation^13^,^14^, we next determined whether blockade of hippocampal CaMKs activity (Fig. 4a) could prevent the effect of novelty on memory persistence. Bilateral infusions of the CaMKs inhibitor KN-93 intra-CA1 region of the dorsal hippocampus^15^, around 11 h after IA training to rats that were not exposed to spatial novelty, did not modify the retention scores in a 7-day test session (Fig. 4b). However, the infusion of KN-93 into the CA1 region of the dorsal hippocampus 15 min before exposing rats to a novel OF 11 h after IA training, blocked the retrograde amnesic effect of OF exposure (Fig. 4b, p < 0.01, compared to Veh + OF rats; Newman-Keuls test after ANOVA, n = 12–13).

## Discussion

Anxiety disorders are among the most prevalent psychiatric illnesses^16^. They are conceptualized as disorders of emotional learning processes^17^ or fear regulation, in which behavioural avoidance is a central feature. The idea of modifying memory processing as a treatment for fear-related disorders is not new. The moment when an aversive experience occurs is the first opportunity to interfere with the formation of a fear-motivated memory. For example, modulation of opioid systems has proved to be effective in PTSD^18^. On the other hand, blocking memory formation after trauma with β-adrenoceptor blockers has yielded mixed results^19^. In addition, early behavioural intervention after a fearful experience in rodents lead to conflicting findings^20^,^21^. In recent years there has been some improvement in the attenuation of fear-related memories by manipulating memory reconsolidation and extinction processes. However, extinction-based exposure therapy might have limited efficacy^1^. Immediate or delayed extinction procedures induce a reduction in aversive memories that are context-dependent and short-lived^2^,^3^. Several reconsolidation experiments revealed that memories become increasingly resistant to pharmacological manipulations as they grow older^22^,^23^, or that postreactivation induced-amnesia recovers over time^24^,^25^. On the other hand, a successful attempt to attenuate fear memories in rats and humans was obtained using a novel behavioural design involving a mixed reconsolidation-extinction procedure^4^,^5^, (but see ref. 6).

Our results demonstrated that a simple behavioural intervention at the critical time window after training attenuates the persistence of fear-motivated memory storage in rats in a time-dependent manner. The amnesic effect of a new learning on previously encoded material is known as retroactive interference (RI). It has been suggested that there are two types of RI that produce forgetting, named diversion and similarity RI. While the former affects consolidation, the latter affects retrieval^26^,^27^. Our present findings represent the first example of a behavioural RI paradigm that successfully impairs LTM persistence in a critical time window many hours after a learning experience. Using this protocol, we expanded the time period in which memory formation and storage can be modified. Moreover, we described that the event that induces the effect is a novel spatial exploration that engages the activation of hippocampal CaMKs. Given that high doses of KN-93 could inhibit some CaMK isoforms^28^, we cannot ascertain which of the isoforms are involved in this effect.

The delayed RI of fear memory persistence caused by novel OF exposure observed in the present study was not due apparently to a deficit in IA memory retrieval. In fact, animals normally expressed fear memory two days after IA training. Also, exposure to the OF 8 or 23 h after IA training left the expression of 2 and 7 day-old fear memories intact. Moreover, spatial novelty enhances, but does not reduce, IA memory retrieval when novelty is close to the testing session^12^. Stress is an unlike factor involved in the deleterious effect of novelty on IA memory maintenance since it has been recently shown that exposure to stress around 12 h after training did not impair but rather enhanced contextual fear memory persistence^29^. We cannot totally rule out that novelty-induced impairment of memory retention might represent a type of “behavioural metaplastic” change^30^. However, behavioural metaplasticity refers to a modification of a behavioural process (in our case the IA training session) by a prior event and not by a subsequent experience as it occurs in our study (an exposure to an OF several hours after training).

Our results provide further evidence showing that memory is highly dynamic and influenced by other events occurring around and beyond the learning to be remembered^31^. In that sense, the strength of training and the type of -and time when- behavioural manipulations are experienced before or after acquisition, will determine the promoting or interfering effects on memory processing^32^,^33^,^34^,^35^,^36^,^37^,^38^,^39^,^40^,^41^,^42^,^43^,^44^. A possible molecular mechanism underlying this effect is based on the behavioural tagging hypothesis which proposes that memory formation and persistence depend on synthesis of new proteins that will be used at specific substrates (tagged sites) in order to establish the memory trace^31^. On this regard, we have recently found that a weak IA training that generates a short-lasting LTM of a couple of days would create a maintenance-specific tag while proteins necessary for memory persistence would be provided by a close-in-time novel experience^44^. This mechanism could explain interference between two or more memory traces. When the amount of proteins is insufficient for capture at multiple tagged sites, a competition for these resources would develop and, as a consequence, one of the memory traces might be impaired. Some studies showed that plasticity at specific synapses and memory formation for particular experiences are, at least in part, the result of competition for available resources^37^,^43^,^45^,^46^,^47^,^48^. Therefore, we suggest that the deleterious effect of the exposure to a spatial novelty on IA-memory persistence is probably due to the “competition maintenance” between tagged sites for the available proteins induced by the two learning tasks. Recently it was showed that dopamine D1/D5 receptor regulates synaptic cooperation and competition in hippocampal neurons^49^. The authors suggested that both, the modulating effect of dopamine on protein synthesis and the number of sites tagged by different stimuli, will interact so that the synapses will cooperate or compete during formation of stable memory traces. Moreover, the authors demonstrated that ERKs are involved in the effect of dopamine D1/D5 receptor agonist on synapse cooperation. However, there is less information regarding possible molecular substrates for the tag. In our study, we showed that CaMKs are involved in the competitive effect of spatial novelty over the persistence of IA memory. We speculate that inactivation of CaMKs may impair the setting of tagged sites induced by OF exposure.

Our finding is utterly promising because it opens a new avenue of research in the control of fear memories. It would be rather difficult to know when a traumatic event is going to take place in our daily life. However, targeting a treatment for fear-related memory disorder many hours after the aversive experience occurred would be a possible intervention to dampen memory. In conclusion, we show that it is possible to attenuate the durability of fear memories by acting on a permissive and restrictive late memory consolidation window (12 h post training). At that time point, and without the need administer any drug^50^,^51^ or to subject animals to a retrieval session^5^, the exploration to spatial novelty is sufficient to reduce a long-lasting fear memory.

## Methods

### Subjects

Experiments were conducted in male Wistar rats from the vivarium of the University of Buenos Aires (Buenos Aires, Argentina) weighting 230–260 g and 2–2.5 months old. Animals were housed five to a cage and kept at a constant temperature of 22 °C, with water and food *ad libitum*, under a 12 h light/dark cycle (lights on at 7:00 A.M.). Each animal was used only for one experiment. Experimental procedures followed the guidelines of the USA National Institutes of Health Guide for the Care and Use of Laboratory Animals and were approved by the Animal Care and Use Committees of the University Buenos Aires (CICUAL).

### Inhibitory avoidance

Animals were handled once a day for two days and then trained in inhibitory avoidance (IA) as described previously^7^. Briefly the apparatus was a 50 × 25 × 25 cm opaque acrylic box whose floor was a grid made of 1 mm caliber stainless steel bars. The left end of the grid was covered by a 7 cm wide, 5.0 cm high platform. During the handling session animals were manipulated in the same way they were during intracerebral infusions (see below). Briefly, they were grasped by hand and slightly restrained in the lap or the arm of the investigator. During the second day of this manipulation in most animals there were no evident signs of stress. For training, animals were gently placed on the platform and, as they stepped down onto the grid, received a single 3 sec, 0.7 mA scrambled foot-shock. Latency for stepping down onto the grid with all four paws was measured. Rats were tested for retention either at 2 days or 7 days after training, depending on the experiment. The test session was similar in all respects to the training session except that the footshock was not given, and the latency was evaluated for a maximum of 300 seconds. All animals were tested only once. Training was always performed between 8:30–9:30 AM. For each experiment the number of animals in each group is detailed in the Results section.

### Open Field

The open field (OF) was a 50 cm high, 50 cm wide, and 39 cm deep arena with black plywood walls and a brown floor divided into nine squares by black lines. The number of line crossings and rearings was measured manually during each minute, in a 5 min test session. The decrease of these parameters is considered an index of spatial habituation^52^.

### Surgery

Sixty rats were bilaterally implanted under deep ketamine/xylacine anesthesia (80 and 5 mg/kg, respectively) with 22-g guide cannulae aimed to dorsal CA1 region of the hippocampus (AP −4.3 mm, LL ± 3.0 mm, DV 3 mm). Coordinates were based on Paxinos and Watson atlas^53^. Cannulae were fixed to the skull with dental acrylic. Obturators were then inserted into the cannulae to prevent blockage, with the same or less length of the cannulae. At the end of surgery, animals were injected with a single dose of meloxicam (0.2 mg/kg) as analgesic and gentamicin (2.5 mg/Kg) as antibiotic.

### Drug infusions

After recovery from surgery (5–7 days), rats were trained in IA and 10.45 h after training received a bilateral infusion of either saline, or the CaMK inhibitor, KN-93 (6 μg/side)^15^. The volume infused was 0.8 μl/side and the infusion rate was 1 μl/min. Injectors were left in place for an additional minute following infusion before they were removed carefully to minimize backflow. Thus, the entire infusion procedure took ~4 min. For intracerebral infusions, 30-Gauge needles connected to Hamilton syringes were used. Infusions were delivered through a needle extending 1 mm beyond the tip of the guide cannula. During the procedure, the animals were slightly restrained with the hands, without provoking any evident stress.

### Cannula placement

To check cannula placement, 24 h after the end of the behavioural procedures, animals were deeply anesthetized and killed by decapitation 15 min later, and histological localization of the infusion sites was established using a binocular magnifying glasses. Coordinates were based on Paxinos and Watson atlas^53^. Schematic representation of rat brain sections showing the approximated extension of the area (gray) reached by the infusions of 0.8 μl of methylene blue in the CA1 region of the dorsal hippocampus is shown in Fig. 4a. Infusions spread about 1.5 mm3 and were found to be correct in 53 out of 60 animals. Cannula placements were exactly as in several previous papers^7^,^9^,^44^,^47^.

### Data analysis

In all behavioural experiments statistical analysis was performed by unpaired Student’s t test or, when required, one-way ANOVA followed by Newman–Keuls multiple comparison test, comparing mean step-down latencies of the OF/drug-treated groups and control/vehicle-treated groups at each time point studied. Data in the bar graphs are presented as mean ± SEM.

## Additional Information

**How to cite this article**: Katche, C. *et al*. Novelty during a late postacquisition time window attenuates the persistence of fear memory. *Sci. Rep.* **6**, 35220; doi: 10.1038/srep35220 (2016).
